# Monocarborane cluster as a stable fluorine-free calcium battery electrolyte

**DOI:** 10.1038/s41598-021-86938-0

**Published:** 2021-04-06

**Authors:** Kazuaki Kisu, Sangryun Kim, Takara Shinohara, Kun Zhao, Andreas Züttel, Shin-ichi Orimo

**Affiliations:** 1 Advanced Institute for Materials Research (WPI-AIMR), Tohoku University, Katahira 2-1-1, Aoba-ku, Sendai, 980-8577 Japan; 2 Institute for Materials Research (IMR), Tohoku University, Katahira 2-1-1, Aoba-ku, Sendai, 980-8577 Japan; 3 Laboratory of Materials for Renewable Energy, École Polytechnique Fédérale de Lausanne (EPFL), Valais/Wallis, Rue de l’Industrie 17, 1950 Sion, Switzerland

**Keywords:** Batteries, Energy, Energy science and technology, Energy storage, Batteries

## Abstract

High-energy-density and low-cost calcium (Ca) batteries have been proposed as ‘beyond-Li-ion’ electrochemical energy storage devices. However, they have seen limited progress due to challenges associated with developing electrolytes showing reductive/oxidative stabilities and high ionic conductivities. This paper describes a calcium monocarborane cluster salt in a mixed solvent as a Ca-battery electrolyte with high anodic stability (up to 4 V vs. Ca^2+^/Ca), high ionic conductivity (4 mS cm^−1^), and high Coulombic efficiency for Ca plating/stripping at room temperature. The developed electrolyte is a promising candidate for use in room-temperature rechargeable Ca batteries.

## Introduction

Li-ion batteries with high energy densities are indispensable in applications such as portable electronic devices, electric vehicles, and grid-scale storage. However, current Li-ion battery technologies are approaching their theoretical energy density limits. In addition, there are critical challenges associated with natural abundance, cost, and safety^1^. A plausible solution to these issues is to use divalent batteries based on Ca or Mg metal anodes, because these elements are the fifth and seventh most abundant in Earth's crust, respectively. In particular, Ca has a low reduction potential (− 2.87 V vs. standard hydrogen electrode (SHE)), similar to that of Li (− 3.04 V vs. SHE) but much lower than that of Mg (− 2.37 V vs. SHE). Furthermore, these divalent metals offer higher volumetric capacities (Ca: 2073 mAh cm^−3^; Mg: 3833 mAh cm^−3^) than Li^2–5^. Thus, the cell voltage and energy density of Ca batteries are expected to be comparable to and higher than those of Li-ion and Mg batteries, respectively. Moreover, as Ca^2+^ (1.12 Å) has a larger ionic radius than that of Mg^2+^ (0.72 Å), its charge polarization is reduced, and this ion softness tends to form more-covalent bonds with host anions, which may lead to improved ion transport and diffusion in electrolyte and cathode materials^3, 6^. Therefore, rechargeable Ca batteries that exhibit not only the advantages of cost effectiveness and abundance but also the battery performances are attractive candidates for post-Li-ion battery technologies^2, 6–8^.

Among the main challenges related to Ca-battery technology is a lack of suitable electrolytes for reversible Ca metal plating/stripping at room temperature^9^. Non-aqueous Ca electrolytes comprising conventional salts in aprotic solvents are fundamentally incompatible with Ca metal anodes because the passivating films that form on anode surfaces prevent Ca ion transport^10^. In the past few years, intensive research has been pursued to develop new electrolytes capable of reversible Ca plating/stripping. In 2016, reversible Ca plating/stripping was first reported in an electrolyte of Ca(BF_4_)_2_ in ethylene carbonate/propylene carbonate at 100°C^11^. The deposited product contained not only Ca metal but also CaF_2_, which inhibits Ca diffusion and hinders plating and stripping processes^12^.

More recently, Li et al. and Shyamsunder et al. simultaneously demonstrated that room-temperature reversible Ca plating/stripping using an electrolyte of Ca[B(hfip)_4_]_2_ (hfip = hexafluoroisopropyloxy) in 1,2-dimethoxyethane (DME) was possible with impressive anodic stability (> 4.0 V)^13, 14^. The [B(hfip)_4_] anion is known to be a weakly coordinating anion with weak anion–cation interactions, thereby favouring ion association and higher conductivities^15, 16^. Despite its excellent electrochemical performances, this electrolyte can intrinsically suffer from the same issue of CaF_2_ formation as the Ca(BF_4_)_2_ electrolyte. Wang et al. proposed Ca(BH_4_)_2_ in tetrahydrofuran (THF) as a fluorine-free electrolyte system, which showed a Coulombic efficiency of 95% during Ca plating/stripping on an Au electrode^17^. Although this electrolyte is compatible to Ca metal, the anodic stability is only 2.4 V vs. Ca^2+^/Ca because of the reducing nature of BH_4_^−^ anions^18, 19^. Thus, it is desirable to find a fluorine-free Ca electrolyte system that delivers high electrochemical performance, i.e. a wide electrochemical potential window, reversible Ca plating/stripping stability, and high ionic conductivity at room temperature.

Herein, we propose a fluorine-free Ca electrolyte using calcium monocarborane (CMC or Ca[CB_11_H_12_]_2_), which shows a wide electrochemical potential window up to 4 V vs. Ca^2+^/Ca and high conductivity of 4 mS cm^−1^, in addition to supporting reversible Ca metal plating/stripping at room temperature. The monocarborane cluster anion ([CB_11_H_12_]^−^) is a type of complex hydride anion, which is known as a weakly coordinating anion^20–23^. Moreover, due to its high reductive and oxidative stability, it allows for a wide potential window and shows excellent stability against metal anodes such as Li, Na, and Mg^24–29^. These led to the idea that an electrolyte with the [CB_11_H_12_]^−^ anion could also be highly compatible with Ca batteries. However, a design that incorporates a monocarborane cluster anion into a Ca electrolyte has not been proposed. In fact, the results of this study reveal that electrolytes prepared by simply adding a CMC salt to single DME or THF solvents show poor solubility and deliver insufficient electrochemical performance. Hence, we found that a DME/THF mixed solvent shows high solubility for the CMC salt and delivers excellent electrochemical performances in Ca batteries.

## Methods

The preparations and handling of air-sensitive materials were conducted under a dry Ar atmosphere using a glovebox and Schlenk techniques.

### Synthesis of CMC electrolytes

Hydrated CMC (CMC·nH_2_O) and anhydrous CMC were synthesised via ionic exchange and heat treatment^30, 31^. First, Cs[CB_11_H_12_] (2.759 g, 10 mmol, Katchem Ltd.) was converted into the corresponding acid [H_3_O][CB_11_H_12_] through ion exchange(acidic form of Ambarlite IR120B, 20 mL). Aqueous Ca[CB_11_H_12_]_2_ was prepared by neutralising [H_3_O][CB_11_H_12_] with excess CaCO_3_(1.501 g, 1.5 eq, FUJIFILM Wako Pure Chemical Co.). Solvent removal yielded hydrated CMC, which was further dried under vacuum (< 8 × 10^−4^ Pa) at 433 K for 10 h to obtain CMC^32^. DME (Sigma-Aldrich), THF (Sigma-Aldrich), DME/THF mixed solvent, diglyme, and triglyme were stored over 3-A molecular sieves prior to use, yielding measured water levels of < 10 ppm. To prepare Ca electrolytes, CMC was dissolved in a volumetric flask with appropriate amounts of each solvent to achieve the desired or saturated concentration. The molar concentration of the electrolyte is based on the molar mass of CMC.

### Physicochemical characterisations of the CMC electrolytes

The Ca, B, and residual Cs contents of the compounds were determined using inductively coupled plasma-optical emission spectrometry (ICP-OES) and inductively coupled plasma mass spectrometry (ICP-MS). Nuclear magnetic resonance (NMR) spectra (Bruker Avance II spectrometer) were obtained at 7.05 T for the ^1^H and ^11^B nuclei. All samples were prepared using aceton-*d6* (Sigma-Aldrich) as the solvent. The vibrational modes of complex anions were characterised by Raman spectroscopy (DXR, Thermo Scientific). Prior to performing scanning electron microscopy (SEM) with energy-dispersive X-ray spectroscopy (EDS) analysis, all the samples were loaded in air-tight sample holders to prevent any exposure to ambient conditions during sample transfer. For the differential scanning calorimetry (DSC) measurements, samples weighing 4.02 mg were transferred through the glovebox and measurements were conducted under Ar flow. After loading the samples in the DSC instrument, a heating programme from T = 303 to T = 773 K with a heating rate of dT/dt = 2 K min^−1^ was started. A return programme for cooling was started sequentially at a cooling rate of dT/dt =  − 2 K min^−1^. The saturated concentrations of CMC in DME, THF, and the DME/THF mixture were determined by performing ICP-OES measurements. The water contents were measured using a convertible Karl Fischer moisture meter (CA-200, Mitsubishi Chemical Analytech Co.,Ltd.).

### Electrochemical analyses and battery tests

Disc-shaped working electrodes composed of Au, Pt, Cu, and SUS (with diameters of 8.0 mm) and the counter and reference comprising Ca (with sizes of diameters of 10.0 mm and diameters of 5.0 mm, respectively) were extensively polished until a metallic lustre was achieved before each use. All the electrochemical analyses were performed at room temperature with a stainless-steel electrochemical cell holder. Cyclic voltammetry (CV) was conducted at 20 mV s^−1^ with the voltage ranging between − 0.4 and 4.0 V vs. Ca^2+^/Ca. Ex-situ XRD measurement was performed using an X’PERT Pro diffractometer (PANalytical) with Cu Kα radiation (wavelength λ = 1.5406 Å for K_α1_ and 1.5444 Å for K_α2_).

Sulfur/carbon (S/C) composites were prepared through mechanical milling using elemental sulfur (99.98%, Sigma-Aldrich), KETJEN BLACK (KB), and MAXSORB with a weight ratio of 2:1:1^24, 33^. S/C electrodes were prepared by mixing 80 wt% of the composite and 20 wt% of weight of polyvinylidene difluoride (PVDF) in N-methyl pyrrolidone. The mixture was coated on an etched Al foil (current collector) and dried at 353 K in a vacuum for 12 h. For the battery tests, the S/C electrodes, separator, electrolyte, and Ca metal anode were placed in a stainless-steel electrochemical cell holder. The electrochemical measurements were conducted at a C-rate of 0.1 C at room temperature in the voltage range of 3.2–0.5 V using a battery tester (580 Battery Test System, Scribner Associates).

X-ray photoelectron spectroscopy (XPS) analyses of the pristine S/C electrode and S/C electrode after discharge at 0.5 V were performed using a PHI 5000 VersaProbe III instrument (ULVAC-PHI, Inc.). The conductivities of solid-phase CMC pellets (8 mm) pressed at 120 MPa were measured using the AC impedance method that utilized Au/CMC/Au over a temperature range of 303–423 K, with applied frequencies of 4 Hz to 1 MHz that were produced using a frequency response analyzer (3532–80, HIOKI).

## Results and discussion

**Figure 1:**
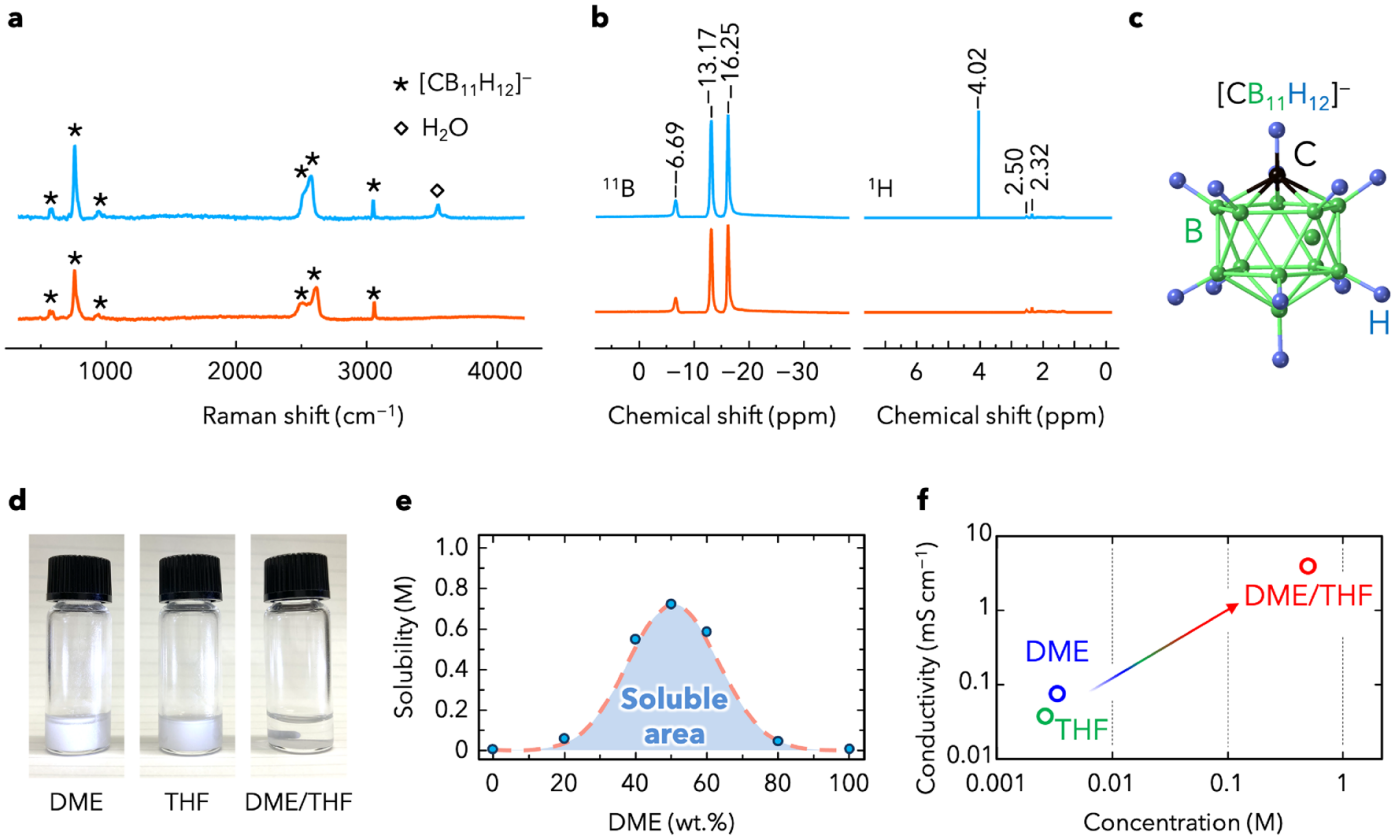
(**a**) Raman spectra of calcium monocarborane (CMC) (orange) and CMC·nH_2_O (blue). (**b**) ^11^B and ^1^H nuclear magnetic resonance (NMR) spectra of CMC (orange) and CMC·nH_2_O (blue). (**c**) Geometry of the [CB_11_H_12_]^–^ anion (black, green, and blue spheres denote C, B, and H atoms, respectively). (**d**) Photographs of CMC dispersed/dissolved in 1,2-dimethoxyethane (DME), tetrahydrofuran (THF), and a DME/THF mixture. (**e**) Solubility diagram of CMC in mixtures of DME/THF with various solvent ratios. (**f**) Relationship between conductivity and CMC solubility (for details, see Table S2 and Fig. S2). The thermal stability is shown in Fig. S4. The conductivity of the solid phase CMC were measured by EIS and shown in Fig. S5.

First, the atomic ratios of Ca, Cs, and B in CMC were determined through ICP-MS and ICP-OES (Table S1). As the Cs/Ca ratio was less than 1/10,000, the Cs salt was almost completely converted into Ca salt. Furthermore, the Ca/B ratio was 1/21.96, which is close to the theoretically determined ratio of 1/22 in CMC (Ca[CB_11_H_12_]_2_). To characterize [CB_11_H_12_]^−^ anion and H_2_O in CMC, Raman spectroscopy measurements and NMR measurements were assessed for CMC and CMC‧nH_2_O (Fig. 1a,b). The Raman spectra of CMC and CMC‧nH_2_O exhibited various deformation vibration modes of [CB_11_H_12_]^−^ below 1200 cm^−1^. In addition, the Raman peak at 3050 cm^−1^ was ascribed to the C–H stretching mode of [CB_11_H_12_]^−^ anion (Fig. 1c)^34^. The Raman peak observed at 3600 cm^−1^ for CMC·nH_2_O, ascribed to the O–H mode of H_2_O^35^, was not detected for CMC, indicating that the hydrated water can be mostly removed from CMC by simply heating akin to the removal of the closo-type Ca complex hydride CaB_12_H_12_^30, 32^. These results are consistent with the ^11^B and ^1^H NMR spectra^28, 36^. To investigate the water content in CMC and CMC‧nH_2_O in detail, the Karl-Fischer titration method was applied for two electrolytes that were prepared from 5 mL of DME/THF (water content is less than 10 ppm) with 20 mg of CMC or CMC‧nH_2_O. The water content of CMC in DME/THF was measured as 15 ppm, while the water content of CMC‧nH_2_O in DME/THF was measured as 1178 ppm. The difference of 5 ppm in the water content would not affect battery operation; this difference can be compensated by the addition of molecular sieves to the CMC electrolyte. Upon the calculation of water content, CMC·nH_2_O can be expressed as CMC·6H_2_O, which is consistent with the heat-treatment-induced weight loss determined by thermogravimetric analysis (Fig. S1).

Ca electrolytes were prepared by dissolving CMC into the weakly coordinating solvents, which enabled the dissociation of Ca salts^37–39^. As the weakly coordinating solvents, THF, DME, and a mixture of DME/THF (1:1, v/v) were selected, and the solubilities of CMC were evaluated by ICP-OES using saturated solutions. Figure 1d shows photographs of 5 mg CMC dissolved or dispersed in 1 mL of solvent. Interestingly, although the solubilities of CMC in DME and THF were very low (< 0.0033 and 0.0026 M, respectively), that in DME/THF was high (> 0.75 M). An investigation of the solubility behaviour of CMC in THF and DME binary mixtures was carried out at room temperature, where Fig. 1e shows the solubility diagram of CMC in DME/THF mixed solvents of various ratios. The highest solubility in the diagram is observed at a solvent ratio of approximately 1/1 (v/v). Similar improvements in solubility have been reported by using mixed solvents for several salts containing cluster-type complex hydride anions, such as Mg(CB_11_H_12_)_2_, Li_2_B_10_Cl_10_, and Li_2_B_12_Cl_12_^29, 40^. One of the possibility for the improved solubility observed in the present study is that the mixed solvents can be functionalized of the monocarborane cluster anion with one or more moieties^41^. The mechanism and role of the mixed solvent can be elucidated through quantum chemistry calculations and X-ray absorption spectroscopy of the electrolyte^42, 43^, which will provide information about the coordination structure surrounding the Ca^2+^ cation and the [CB_11_H_12_]^−^ anion; we plan to conduct such investigations in our future studies. These conductivities were determined using a symmetrical cell with an Au electrode with the cell constant obtained via cell calibration using 0.08% KCl and were used to calculate the conductivity of the electrolyte. The calculated conductivity of 0.5 M CMC/DME/THF (4.0 mS cm^−1^) was greater than those of CMC/DME (0.073 mS cm^−1^) and CMC/THF (0.036 mS cm^−1^), and of the same order as that of the previously reported Ca[B(hfip)_4_]_2_ electrolyte (Figs. 1f, S2, S3 and Table S2)^13^.

**Figure 2:**
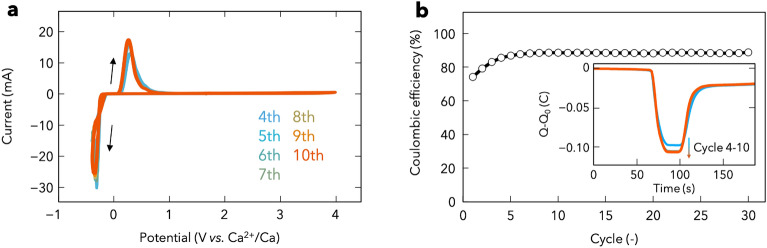
(**a**) Cyclic voltammograms of Ca plating/stripping after conditioning cycles at 20 mV s^−1^ with a three electrode setup using Au as the working electrode and Ca as the reference and counter electrodes at room temperature. Cyclic voltammograms for initial three cycles as conditioning processes are shown in Fig. S6. (**b**) Coulombic efficiency determined from the cyclic voltammograms. Inset: charge balance for cycles 4–10.

Electrochemical studies were conducted using 0.5 M CMC/DME/THF. Ca plating/stripping was performed via CV at 20 mV s^−1^ with three-electrode setup using Ca metal as the reference and counter electrodes, and Au metal as the working electrode. Typical metal plating/stripping behaviour was observed in the cathodic/anodic scans (Fig. 2a). Ca plating commenced at − 220 mV in the cathodic scan, and in the anodic scan, the current began to rise at ∼ 80 mV, indicating the stripping of plated Ca. After conditioning for three cycles (Fig. S6), plating/stripping proceeded with a lower overpotential and improved reversibility. Moreover, the Coulombic efficiency increased to ca. 88% during the initial several cycles and then remained steady over the 30th cycle (Fig. 2b). The insufficient Coulombic efficiency seems to be a result of the partially decomposed CMC/DME/THF electrolyte on the Au electrode and the formation of dead Ca, which is electrically isolated from the electrode. This will be discussed in a different section of this paper along with the SEM/EDS results. Even if the Au working electrode is replaced by an electrode of another metal (e.g., Pt, Cu, and SUS), low overpotential, reversibility, and moderate Coulombic efficiency were still obtained (Fig. S7). On the other hand, the Ca plating/stripping behaviours were not observed when using electrolytes of 0.1 M CMC in diglyme and 0.1 M CMC in triglyme (Fig. S8). Considering the influence of the type of ether solvent and anion coordination on Ca, the incompetency of these electrolytes arises due to the coordination of Ca with diglyme and triglyme being stronger than that of Ca with DME and THF^37^.

**Figure 3:**
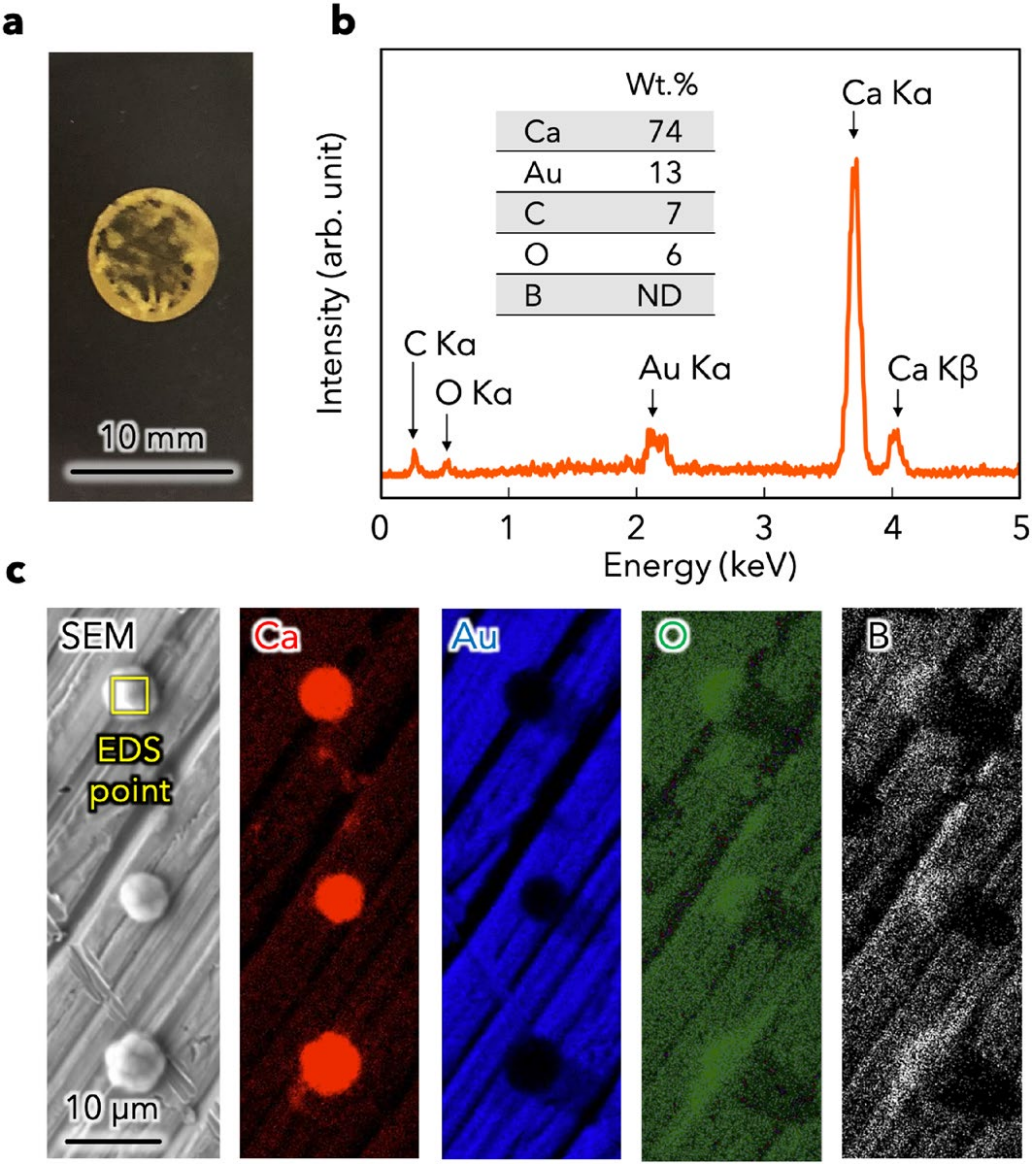
(**a**) Optical image of the Au electrode after a Ca plating process. (**b**) Energy-dispersive X-ray spectroscopy (EDS) profile within the yellow square. (**c**) Scanning electron microscopy (SEM) image of Ca deposits on the Au electrode after Ca plating in a Au | CMC/DME/THF | Ca cell, and EDS maps of Ca, Au, O, and B.

To provide direct evidence for Ca ionic conduction through the CMC/DME/THF electrolyte, we assembled a Ca | CMC/DME/THF | Au cell and deposited Ca on the Au electrode at room temperature. In the disassembled cell, the deposits appeared as black powder on the Au electrode, which was washed with DME/THF (Fig. 3a). The black powder on the Au electrode and separator was removed and collected for XRD measurement, which revealed that the dominant product is Ca metal in the form of α-Ca and β-Ca, along with a small amount of CaH_2_ (Fig. S9). These broad peaks with a low intensity indicate a small size and a low crystallinity for all deposited materials. Then, the morphology of the deposits on the Au electrodes in CMC/DME/THF electrolyte were examined using SEM and EDS, and showed uniformly dispersed spherical particles (Figs. 3b,c, and S10). The spherical particle deposits have a shape similar to that of the Ca deposits prepared in the Ca(BH_4_)_2_–LiBH_4_–THF electrolyte^44^. The EDS profile indicates that the deposits were mainly composed of Ca, O and C with trace amounts of B (Fig. 3b). The large amount of O originated from the highly reactive fresh Ca deposits being briefly exposed to air before the EDS observation (Fig. 3c). The moderate amounts of C and B were likely due to electrolyte reduction at low potentials or residual CMC. The Ca weight ratio in a typical particle was calculated as 84% when contribution of Au content was eliminated. Accordingly, we concluded that the charge carrier in this system is Ca.

In addition, black deposits were also observed on the glass separator after peeling it off of the Au electrode (Fig. S11). The deposits were collected from the glass separator for SEM and EDS analysis by sticking the carbon tape onto the glass and then peeling it off. The obtained images show that the deposits comprise Ca metal and are relatively larger than those on the Au electrode (Fig. S12). The poor adhesion to the Au electrode indicates that they easily lose contact with the electrode, resulting in the formation of dead Ca. This dead Ca formation and the electrolyte reduction at low potentials could cause the insufficient Coulombic efficiency during the Ca plating/stripping processes.

**Figure 4:**
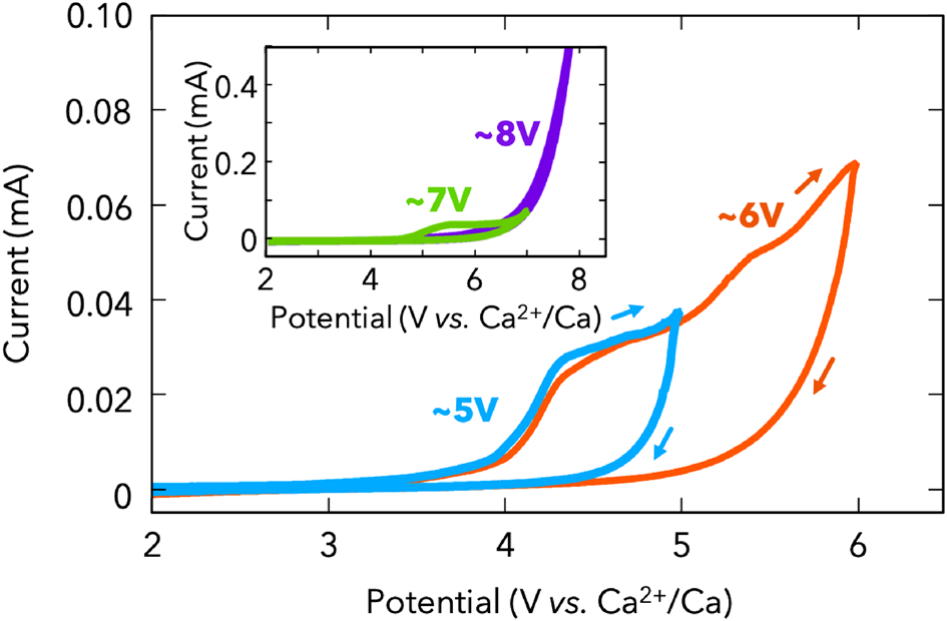
Cyclic voltammograms at 1 mV s^−1^ with different voltage ranges above 5 V (blue), 6 V (orange), 7 V (green), and 8 V (purple). Arrows indicate the sweeping direction during voltammetry. (d) Cycling performance of the Ca | CMC/DME/THF | Ca cell at a current density of 0.02 mA cm^−2^.

To investigate the anodic stability of the CMC/DME/THF electrolyte system, further CV measurements were performed using the Au electrodes with different voltage ranges (Fig. 4). The current density increased at a potential of approximately 4 V, followed by suppression of further electrolyte breakdown and the absence of a significant cathodic current density during the reverse potential sweep even at a higher voltage of ~ 7 V. This finding is consistent with the behaviour observed in electrolytes containing carborane anions with magnesium and tetraethylammonium cations rather than electrolytes containing BH_4_ anions^17, 45^.

**Figure 5:**
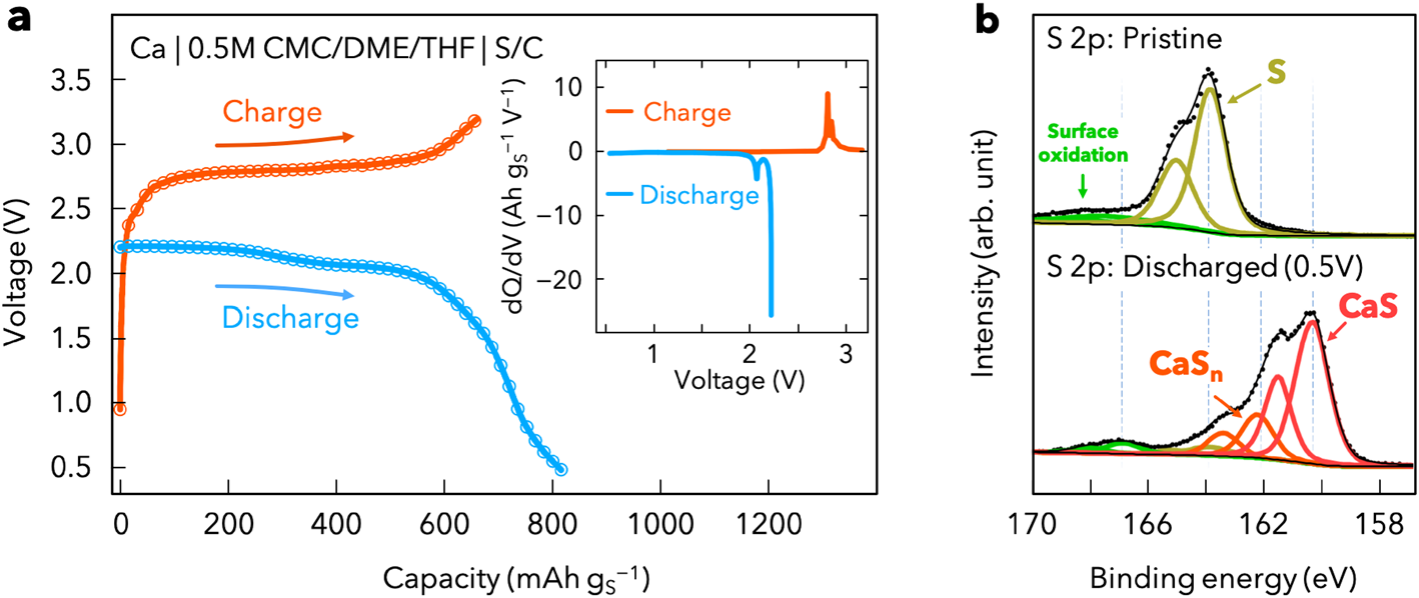
(**a**) Electrochemical performance of the Ca–S batteries with the CMC/DME/THF electrolyte in a voltage window of from 0.5 to 3.2 V; the inset shows differential capacities (dQ/dV), derived from the corresponding charge–discharge curve. (**b**) X-ray photoelectron spectroscopy (XPS) S 2p spectra of the S/C electrodes in the pristine (top) and discharged (bottom) states.

Finally, to investigate the feasibility of the CMC electrolyte, we conducted an exploratory test involving a Ca–S battery, which is a very promising system owing to its high theoretical energy density of 3202 Wh L^−1^^46^. A Ca–S battery, viz. Ca | CMC/DME/THF | S/C was tested with a current density of 167.2 mA g^−1^ (0.1 C per S) at room temperature (Fig. 5a). The initial discharge and charge capacities of the S/C cathode were up to 805 and 750 mAh g^−1^, respectively. Furthermore, it displayed a flat voltage plateau of ~ 2.4 V vs. Ca^2+^/Ca corresponding to the sulfur redox reactions with Ca (Fig. 5a inset)^47, 48^. To obtain information on the conversion reaction of the S/C cathode with Ca, XPS measurements were performed on the pristine electrode and the electrode after discharge. The S 2p spectra of the pristine S/C cathode display the spin–orbit-splitting doublet for elemental S with the S 2p_3/2_ and S 2p_1/2_ peaks at 164.0 and 165.2 eV, respectively (Fig. 5b top). After the electrode discharged to 0.5 V, the S 2p signal spectra can be deconvoluted into three doublet peaks. In addition to peaks of the elemental S, new peaks at 160.3 eV and 162.2 eV attributed to terminal sulfur atom in polysulfides of CaS_x_ (2 ≤ x < 8) and calcium sulfide (CaS), respectively, reflecting the effective conversion of S to sulfides (Fig. 5b bottom). This result demonstrates the practical applicability of the CMC electrolyte for Ca–S batteries at room temperature, with a Ca metal anode.

In summary, we developed a highly stable and efficient fluorine-free Ca electrolyte based on a monocarborane anion, viz. a CMC electrolyte, for room-temperature Ca batteries. CMC salts were successfully prepared via simple cation exchange and heating processes, thus indicating that the synthetic method using an aqueous solution is scalable and very promising from an application perspective. The CMC salt exhibited low solubilities in THF and DME, but high solubility in the mixed solvent of DME/THF (1/1, v/v). The CMC electrolyte at 0.5 M showed the most promising electrochemical performances, viz., a high conductivity, wide voltage window, and reversible Ca plating/stripping behaviour with high Coulombic efficiency. In a feasibility study, we used the CMC electrolyte in a Ca–S battery exhibiting reversible discharge and charge abilities as well as a high capacity of 805 mAh g^–1^, demonstrating that the CMC electrolyte is compatible with a Ca–S battery system. The development of a promising electrolyte candidate based on complex hydrides compatible with Ca batteries will create future opportunities for exploring other related complex hydride compounds as Ca salts^26, 49–52^. In addition, the absence of fluorine and CaF_2_ formation in these materials will intrinsically pave the way for achieving high cyclability in Ca batteries. These findings will contribute toward the development of practical electrolytes for room-temperature rechargeable Ca batteries.

## Supplementary Information

Supplementary Information.
