# Supplementary material for: Monocarborane cluster as a stable fluorine-free calcium battery electrolyte
Source: Sci Rep. 2021 Apr 6;11:7563. doi: 10.1038/s41598-021-86938-0 (PMC8024376; doi:10.1038/s41598-021-86938-0)
Supplement: Supplementary file 1 — Supplementary Information. [file 41598_2021_86938_MOESM1_ESM.pdf]

Supplementary information

Monocarborane Cluster as a Stable Fluorine-Free Calcium Battery Electrolyte

Kazuaki Kisu[1]*, Sangryun Kim[2], Takara Shinohara[2], Kun Zhao[3], Andreas Züttel[3], and Shin-ichi Orimo[1,2]*

[1]Advanced Institute for Materials Research (WPI-AIMR), Tohoku University, Katahira 2-1-1, Aoba-ku, Sendai 980-8577, Japan E-mail: k.kisu@imr.tohoku.ac.jp; orimo@imr.tohoku.ac.jp

[2]Institute for Materials Research (IMR), Tohoku University, Katahira 2-1-1, Aoba-ku, Sendai 980-8577, Japan.

[3]Laboratory of Materials for Renewable Energy, École polytechnique fédérale de Lausanne (EPFL), Valais/Wallis, Rue de l'Industrie 17, CH-1950 Sion, Switzerland

Correspondence and requests for materials should be addressed to

K.K (email: k.kisu@imr.tohoku.ac.jp) or S.O. (email: orimo@imr.tohoku.ac.jp)

Table S1. Calcium, Boron, and Cesium contents based on calcium content calculated by ICP-OES and ICP-MS measurements.

	Theoretical	This work
Calcium content	1	1
Boron content	22	21.96
Cesium content	-	1.5×10^{-5}

Table S2. Solubilities and conductivities of calcium mono-carborane in various solvents and mixed solvent at room temperature

	Solubility / mol L^{-1}	Conductivity / mS cm^{-1}
DME/THF*	0.5	4.0
DME	0.0033	0.073
THF	0.0026	0.036

*The solubility and conductivity of CMC in DME/THF electrolyte are shown as highest conductivity calculated by the tests with different electrolyte concentration as shown in Figure S3.

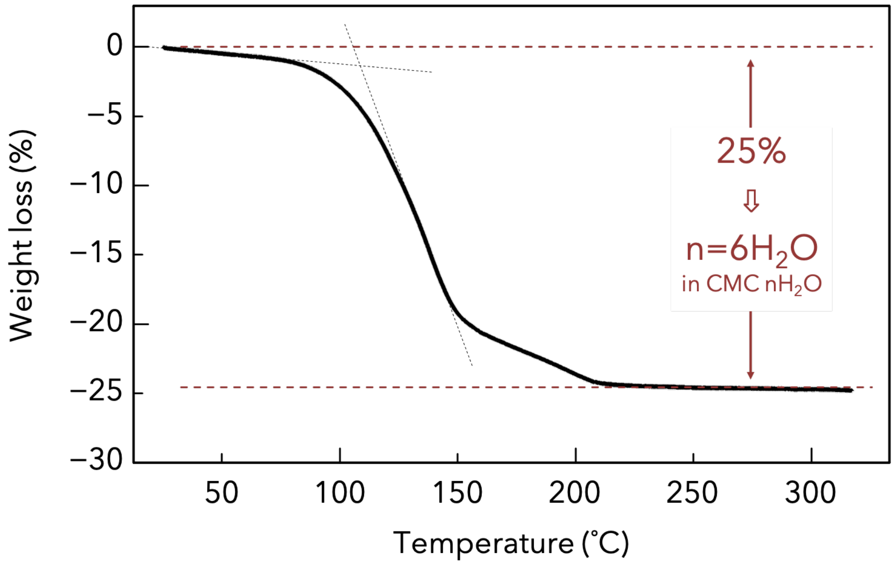

Figure S1. TGA curves for CMC nH_2O at a sweep rate of 1 ℃ min^{-1} from ambient temperature to 320 ℃ under an Ar atmosphere.

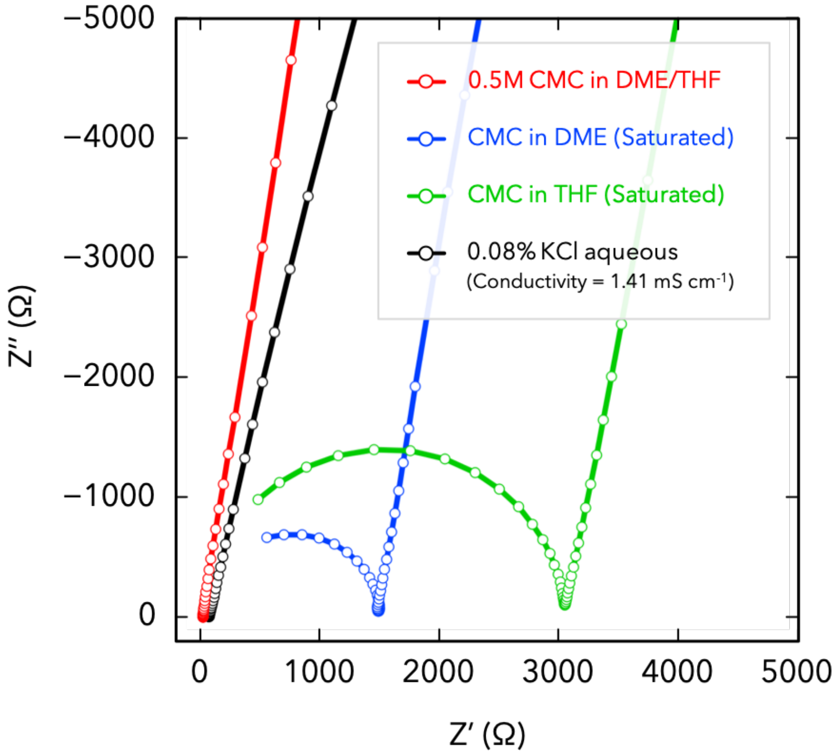

Figure S2. Nyquist plots for Au | Au symmetric cell with different electrolyte of 0.5M CMC in DME/THF (red), CMC in DME (blue), CMC in THF (green), and 0.08% KCl aqueous electrolyte (black).

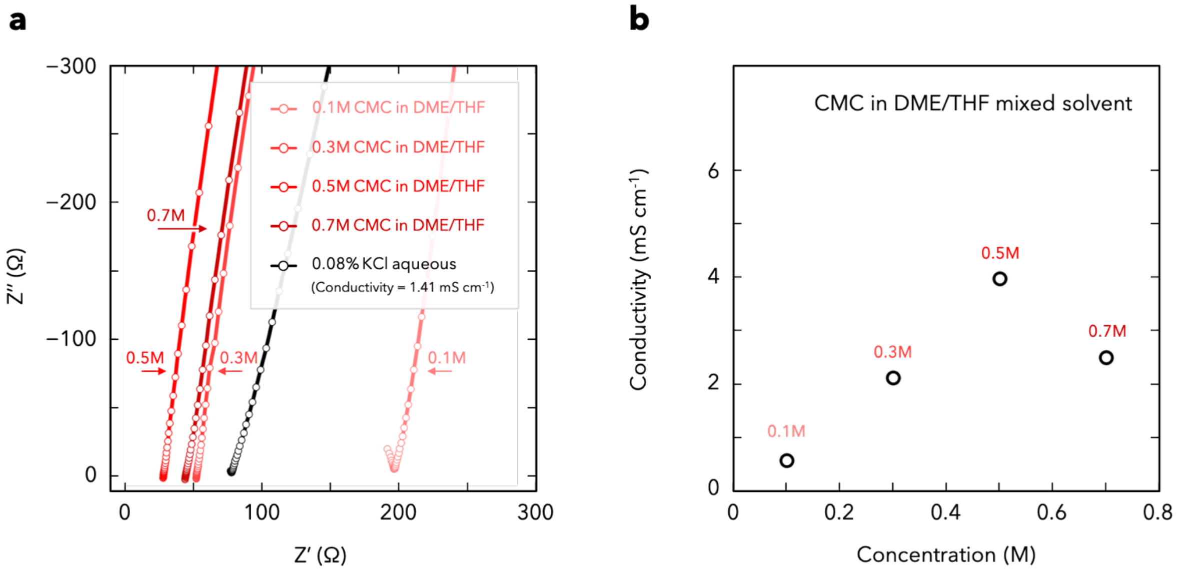

Figure S3. (a) Nyquist plots for Au | CMC in DME/THF | Au symmetric cell with different electrolyte concentration of 0.1M, 0.3M, 0.5M, and 0.7M. (b) Relationship between conductivity and electrolyte concentration.

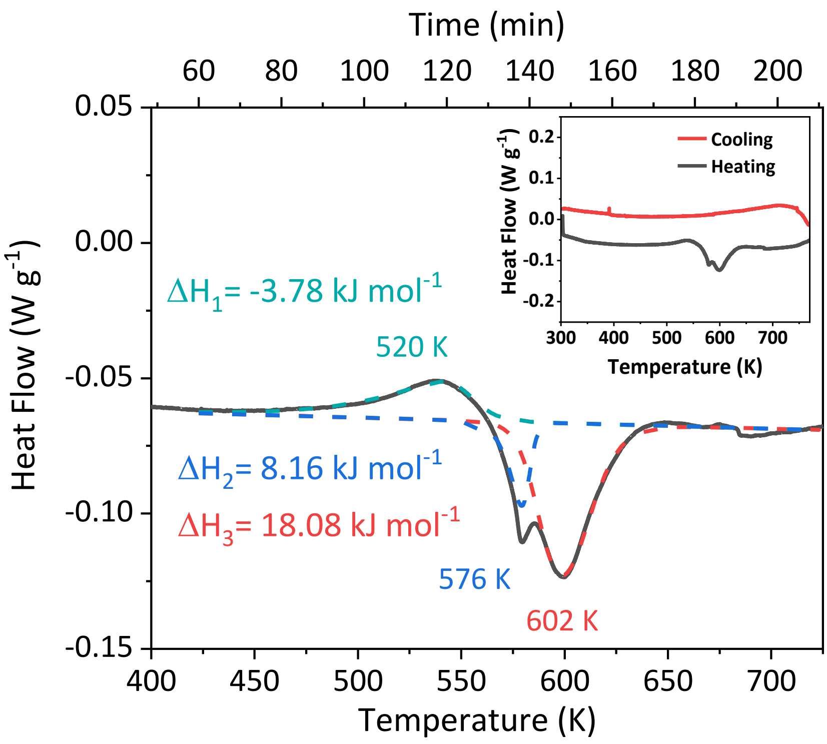

Figure S4. DSC measurement for the CMC and the analyzed peaks and calculated enthalpy. Inserted figure as the return scan from 303-773 K and from 773-303 K.

DSC Analysis. The calculated heat capacity of CMC is 0.062 W g^{-1}, matching with the theoretical heat capacity (3R, where R is gas constant) for solid a tom above room temperature. Above 473 K, a weak exothermic process took place slowly. The peak centered at 520 K and carried the enthalpy of -3.78 kJ mol^{-1}. Two consecutive endothermic peaks showed up at 576 and 602 K, respectively. These two overlapped peaks were deconvoluted and calculated the enthalpy as 8.16 and 18.08 kJ mol^{-1}, respectively. No exothermic peak was observed when cooling from 773 K to room temperature (the inserted figure). This is a sign of no recrystallization taking place, and implies the sample was not melted in the applied heating process. In addition, the measured heat flow of the two endothermic reactions is weak, suggesting the endothermic reactions are unlikely decomposition of the sample. Therefore, the two endothermic reactions could be attributed to two irreversible phase transitions of the [closo-$CB_{11}H_{12}]^-$ anion.[1-4]

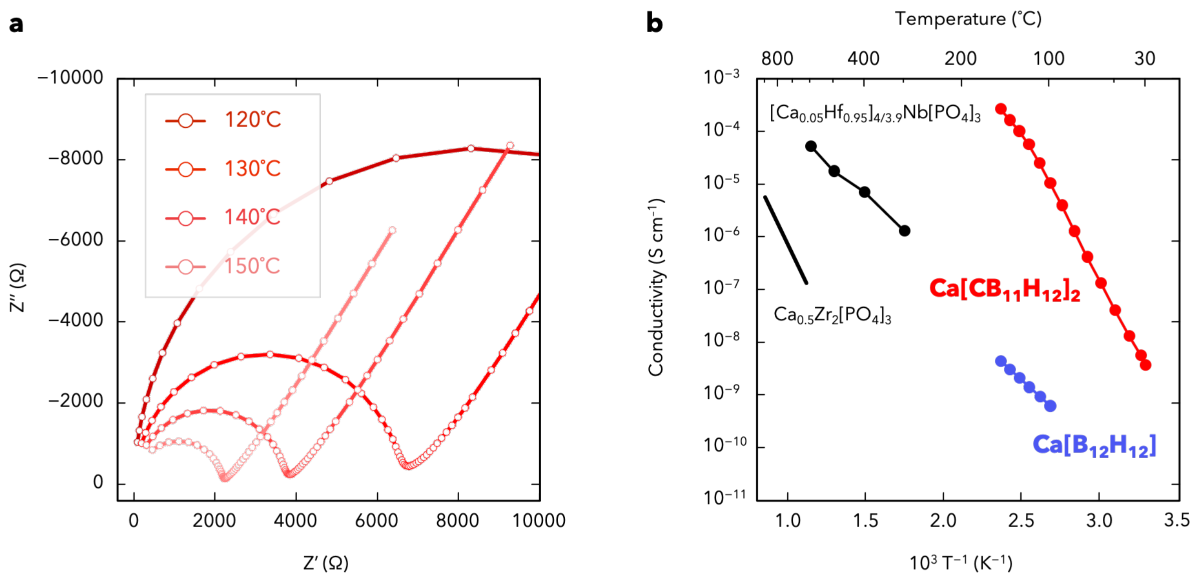

Figure S5. (a) Nyquist plots for the solid phase CMC measured in Au|Au symmetric cell between 120 and 150 °C with applied frequencies of 4 Hz to 1 MHz. (b) Temperature dependence of the conductivity for the solid phase CMC compared to those of previously reported Ca ionic conductor[5] and $Ca[B_{12}H_{12}]$. The sample of $Ca[B_{12}H_{12}]$ was prepared by ionic exchange method[6].

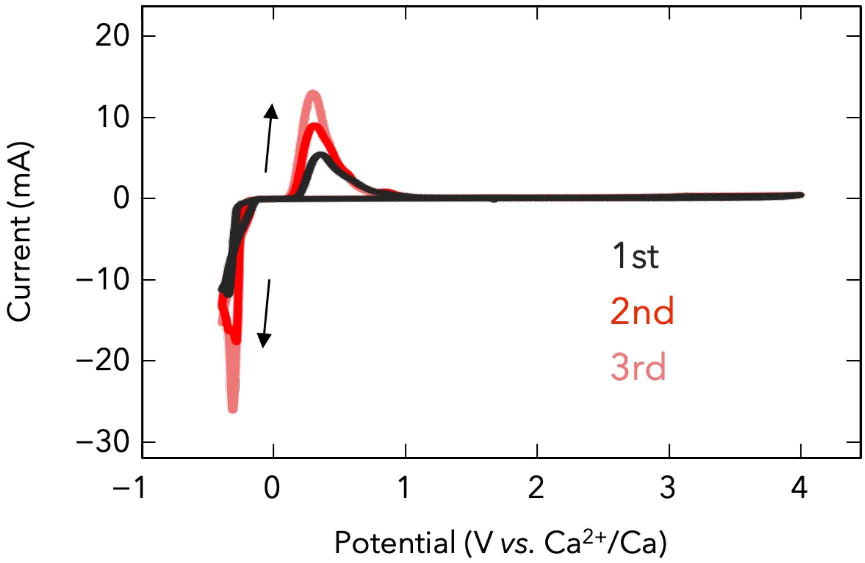

Figure S6. Cyclic voltammograms of Ca plating/stripping for initial three cycles as conditioning processes at 20 mV s^{-1} with a three electrode setup using Au as the working electrode and Ca as the reference and counter electrodes at room temperature.

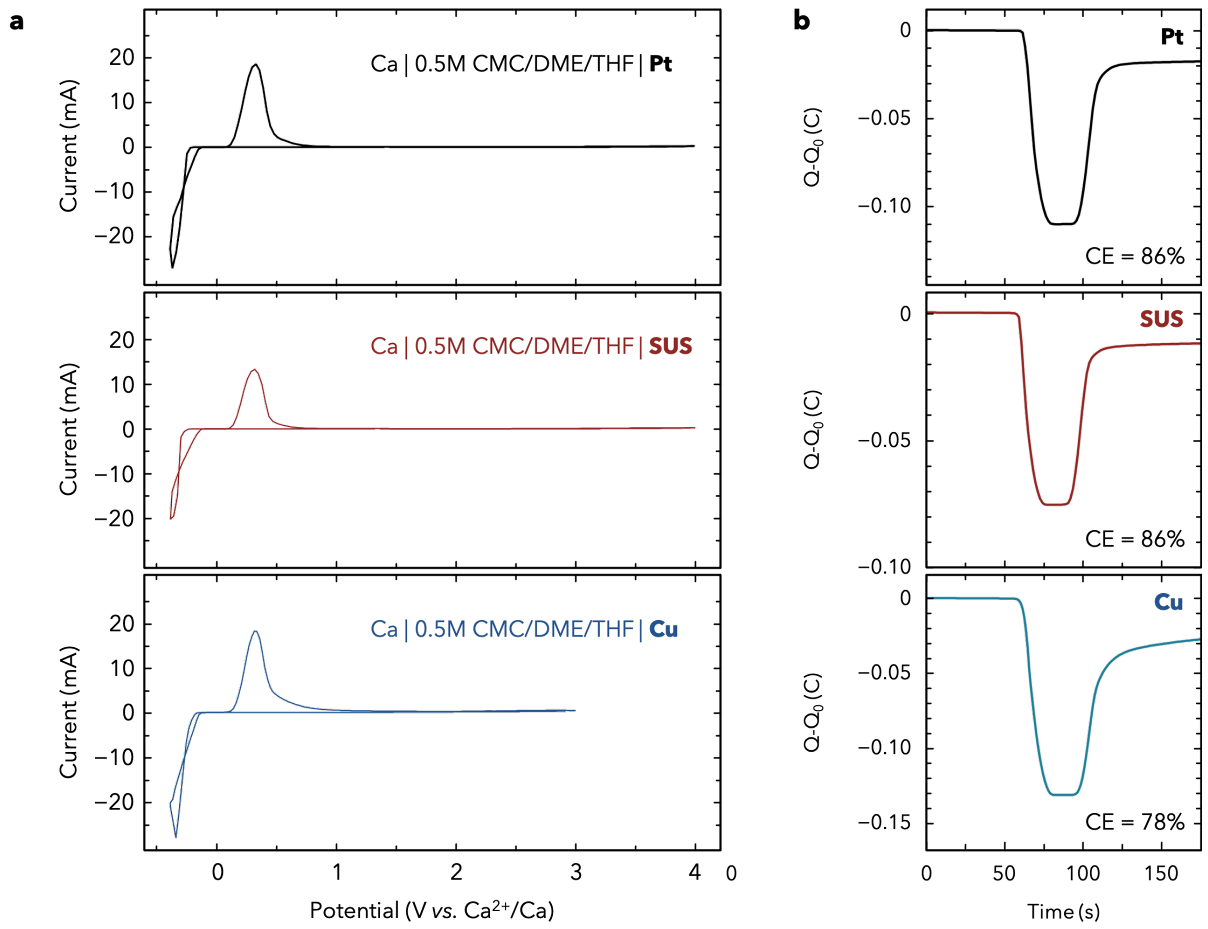

Figure S7. (a) Cyclic voltammograms of Ca plating/stripping in the electrolyte after conditioning cycles at a scan rate of 20 mV s^{-1} with voltage window of −0.4 V and 4.0 V *vs.* Ca^{2+}/Ca with three electrode setup using Pt, SUS, and Cu as a working electrode and Ca as a reference electrode and counter electrode, respectively at room temperature. (b) Coulombic efficiency determined from the cyclic voltammograms.

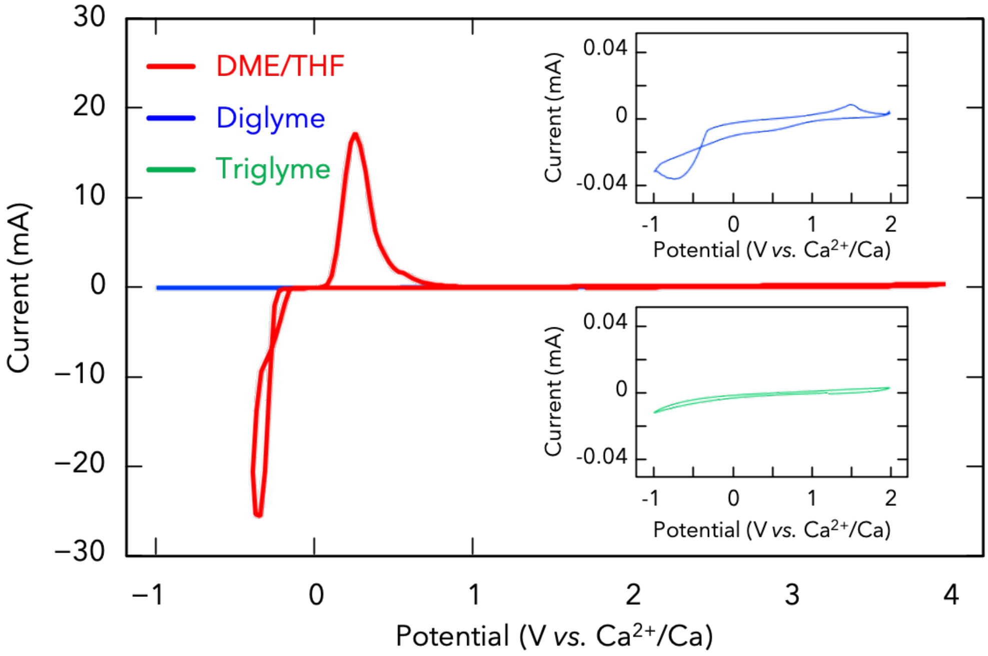

Figure S8. Cyclic voltammograms of Ca plating/stripping in the different electrolytes of 0.5M CMC/DME/THF, 0.1M CMC/Diglyme, and 0.1M CMC/Triglyme after conditioning cycles at a scan rate of 20 mV s^{-1} with different voltage ranges (0.5M CMC/DME/THF: −0.4 V and 4.0 V *vs.* Ca^{2+}/Ca, 0.1M CMC/Diglyme and 0.1M CMC/Triglyme: −1.0 V and 2.0 V *vs.* Ca^{2+}/Ca) using three electrode setup using Au as a working electrode and Ca as a reference electrode and counter electrode, respectively at room temperature.

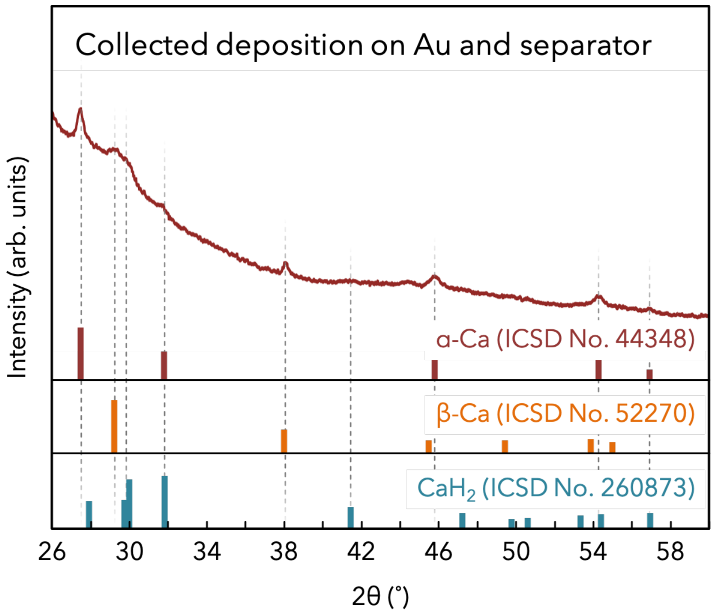

Figure S9. The ex-situ XRD pattern of Ca deposits and reference patterns of Ca alfa (ICSD No. 44348), Ca beta (ICSD No. 52270), and CaH_2 (ICSD No. 260873).

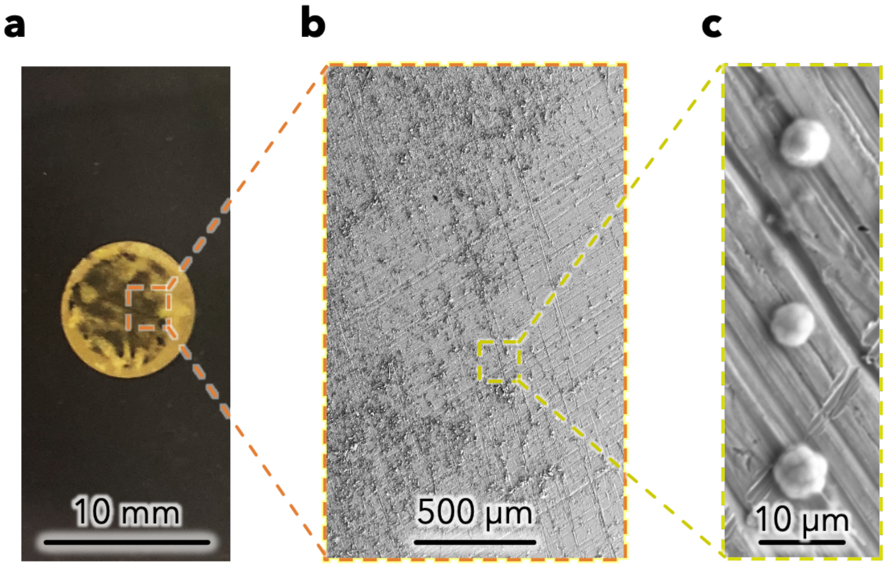

Figure S10. (a) Optical image of the glass separator after Ca plating, (b) Lower magnification scanning electron microscopy (SEM) image of Ca deposits on the Au electrode after Ca plating in a Au | CMC/DME/THF | Ca cell to observe the overall surface condition. (c) Higher magnification SEM image.

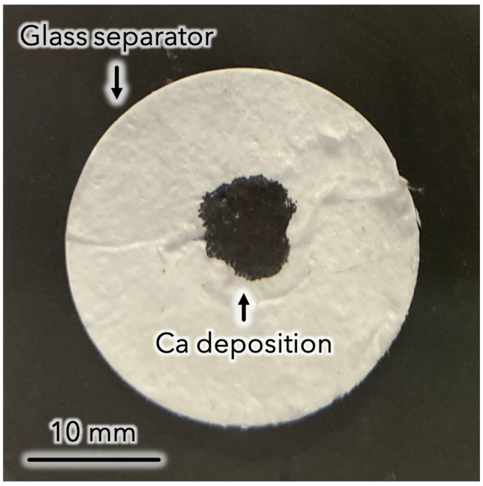

Figure S11. Optical image of the glass separator after a Ca deposition process.

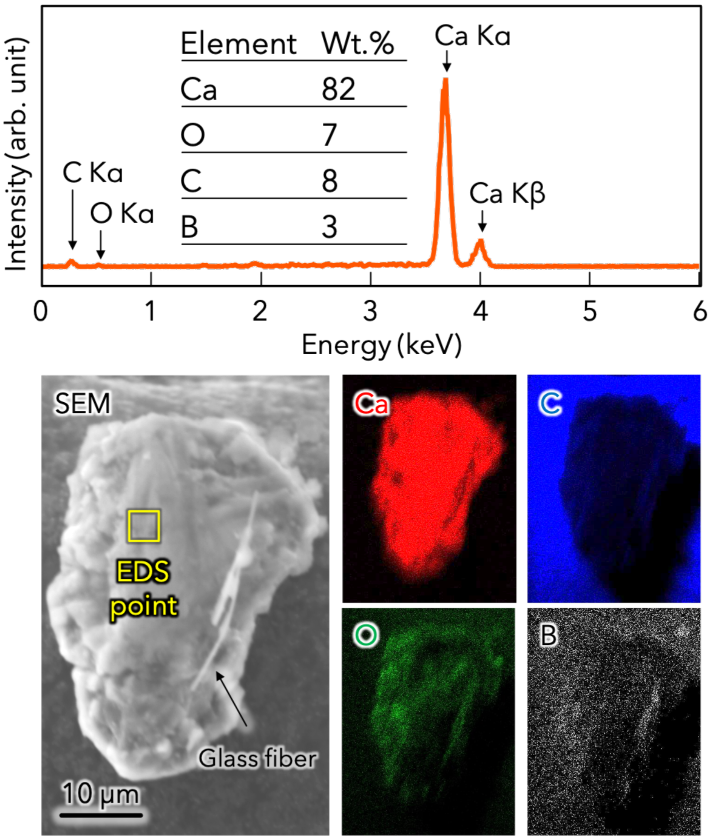

Figure S12. The SEM image and elemental EDS maps of the Ca deposits on carbon tape after Ca plating in a Au | CMC/DME/THF | Ca cell (bottom). The EDX spectrum within the yellow squared area (top).

References

1 Kar, M., Tutusaus, O., MacFarlane, D. R. & Mohtadi, R. Novel and versatile room temperature ionic liquids for energy storage. *Energy Environ. Sci.* **12**, 566-571, https://doi.org/10.1039/C8EE02437E (2018).

2 Körbe, S., Schreiber, P. J. & Michl, J. Chemistry of the Carba-closo-dodecaborate(−) Anion, $CB_{11}H_{12}$. *Chem. Rev.* **106**, 5208-5249, https://doi.org/10.1021/cr050548u (2006).

3 Dymon, J. *et al.* Designing ionic liquids with boron cluster anions: alkylpyridinium and imidazolium [nido-$C_2B_9H_{11}$] and [closo-$CB_{11}H_{12}$] carborane salts. *Dalton Trans*, 2999-3006, https://doi.org/10.1039/b802374c (2008).

4 Romerosa, A. M. Thermal, structural and possible ionic-conductor behaviour of $CsB_{10}CH_{13}$ and $CsB_{11}CH_{12}$. *Thermochim. Acta* **217**, 123-128, https://doi.org/https://doi.org/10.1016/0040-6031(93)85103-G (1993).

5 Lee, W., Tamura, S. & Imanaka, N. New Calcium Ion Conducting Solid Electrolyte with NASICON-type Structure. *Chem. Lett.* **46**, 1486-1489, https://doi.org/10.1246/cl.170634 (2017).

6 Stavila, V. *et al.* Probing the structure, stability and hydrogen storage properties of calcium dodecahydro-closo-dodecaborate. *J. Solid State Chem.* **183**, 1133-1140, https://doi.org/10.1016/j.jssc.2010.03.026 (2010).
